# ﻿A new squat lobster (Crustacea, Decapoda, Munidopsidae) from the western Atlantic with redescription of *Munidopsisexpansa* Benedict, 1902 and several range extensions

**DOI:** 10.3897/zookeys.1248.156346

**Published:** 2025-08-08

**Authors:** Paula C. Rodríguez-Flores, Heather D. Bracken-Grissom, Rafael Lemaitre, Darryl L. Felder, Martha S. Nizinski

**Affiliations:** 1 Department of Invertebrate Zoology, National Museum of Natural History, Smithsonian Institution, 10th and Constitution Ave, NW, Washington, DC 20560, USA National Museum of Natural History, Smithsonian Institution Washington United States of America; 2 Department of Organismic and Evolutionary Biology, Museum of Comparative Zoology, Harvard University, 26 Oxford St., Cambridge, MA 02138, USA Harvard University Cambridge United States of America; 3 Institute of Environment, Department of Biological Sciences, Florida International University, 3000 NE 151st St, North Miami, FL 33181, USA Florida International University North Miami United States of America; 4 Department of Invertebrate Zoology, National Museum of Natural History, Smithsonian Institution, Museum Support Center 4210 Silver Hill Road, Suitland, MD 20746, USA National Museum of Natural History, Smithsonian Institution Suitland United States of America; 5 Department of Biology and Laboratory for Crustacean Research, University of Louisiana at Lafayette, Lafayette, LA 70504, USA University of Louisiana at Lafayette Lafayette United States of America; 6 NMFS National Systematics Laboratory, Smithsonian Institution, P.O. Box 37012, NHB, MRC-153, Washington, DC 20013-7012, USA NMFS National Systematics Laboratory, Smithsonian Institution Washington United States of America

**Keywords:** Barcoding, *COI*, Crustacea, Galatheoidea, Gulf of Mexico, integrative taxonomy, micro-CT scanning, morphology, new record, new species

## Abstract

The western Atlantic Ocean harbors a rich fauna of deep-sea squat lobsters that have been intensively studied during the last two centuries. We revise material recently collected by trawls, ROV and submersibles on several expeditions in the Gulf of Mexico and the Caribbean Sea. Using an integrative taxonomy approach, we describe *Munidopsismessingi***sp. nov.** and redescribe *M.expansa* Benedict, 1902, the latter species known only from a few records. Additionally, we report a new record of *M.turgida* Rodríguez-Flores, Macpherson & Machordom, 2018 for the Gulf of Mexico. This rare species was previously known from only the holotype, collected in the Guadeloupe Islands, Caribbean Sea. We apply micro-CT scanning in the course of morphological illustrations and report barcode data as available.

## ﻿Introduction

Squat lobsters are an extremely diverse group of anomuran crustaceans that inhabit broad geographic and bathymetric ranges (Schnabel et al. 2011). The genus *Munidopsis* Whiteaves, 1874 is recognized as one of the most diverse genera within Decapoda (De Grave et al. 2023) and currently comprises 284 recognized species distributed in all oceans, most at depths ranging from the continental slope to greater than 5000 m (Baba et al. 2008; DecaNet 2025). Measured species diversity within the genus has risen steadily, driven in part by expanded deep-sea exploration but also by greater access to molecular genetic tools. Molecular phylogenetic evidence suggests that the genus is polyphyletic (Ahyong et al. 2011; Rodríguez-Flores et al. 2023).

While the Indo-West Pacific Ocean (IWP) hosts the highest diversity of species of *Munidopsis*, the western Atlantic also supports a diverse representation of the genus (Baba et al. 2008; Felder et al. 2009; Schnabel et al. 2011; Poupin and Corbari 2016). Early contributions by A. Milne-Edwards (1880) and Milne-Edwards and Bouvier (1897) revealed the high diversity of species of *Munidopsis* inhabiting the deep sea in the Gulf of Mexico, the Caribbean Sea and the Atlantic coast of the United States. The unpublished PhD thesis by Barbara Shuler Mayo (Mayo 1974) critically advanced the taxonomy of the genus by providing geographic and bathymetric distribution patterns, as well as a key to the species. These studies laid the foundation for additional taxonomic work in the region (Benedict 1902; Boone 1927; Chace 1942; Pequegnat and Pequegnat 1970, 1971; Mayo 1972; Gore 1983; Baba and Camp 1988; Pequegnat and Williams 1995; Baba et al. 2008; Felder et al. 2009; Vázquez-Bader et al. 2014; Macpherson et al. 2016; Rodríguez-Flores et al. 2018, 2024) that resulted in a significant increase in the number of recognized species.

Recent expeditions to the Gulf of Mexico, the Atlantic coast of Florida, and Curaçao (Caribbean Sea) have provided additional specimens of *Munidopsis*. This material, collected using trawls, remotely operated vehicles (ROVs) and manned submersibles, included several specimens of taxonomic interest, which we address in the present work. Based on morphological and molecular evidence, we describe a species new to science collected off the Atlantic coast of Florida and the Caribbean. As this new species is closely related to *M.expansa* Benedict, 1902, additional specimens of that species were comparatively examined and illustrated. We also report the first collection of *M.turgida* Rodríguez-Flores, Macpherson & Machordom, 2018 from the Gulf of Mexico. This rare species was previously known only from the holotype collected in the Guadeloupe Islands, Caribbean Sea.

## ﻿Methods

### ﻿Specimen collection

**Figure 1.**
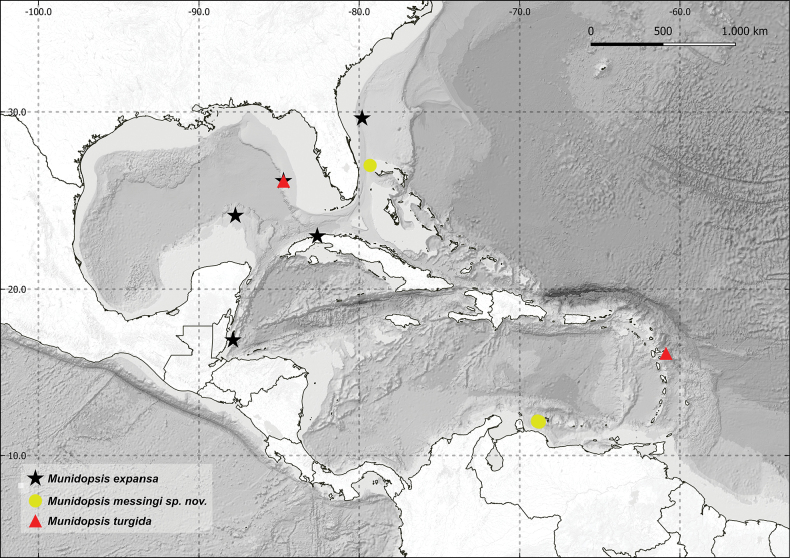
Map depicting locations for species of *Munidopsis* treated in present study of the Gulf of Mexico, Caribbean, and adjacent Atlantic waters off Florida.

Specimens were collected using ROV, submersibles, and trawls between 2003 and 2015 during expeditions or projects in the western Atlantic at depths ranging between 253 and 525 m (Fig. 1). These specimens are deposited at the Smithsonian National Museum of Natural History (**USNM**), Washington DC. Additional historical material housed at USNM, as well as the invertebrate collection at the Museum of Comparative Zoology, Harvard University (**MCZ**), Cambridge, MA and the Florida International Crustacean Collection (**FICC**) at the Florida International University (**FIU**), Miami, FL, were also examined. Abbreviations for fieldwork expeditions and collecting methods account as: U.S. Geological Survey Lophelia II expedition (**USGS-LOPH II**), remotely operated vehicles (**ROVs**), Deep Reef Observation Project (**DROP**).

### ﻿Morphological examination

Specimens were examined using a Leica MZ 12.5 stereomicroscope at the Museum Support Center, Smithsonian Institution. Drawings were made using a camera lucida and were digitized using a Wacom One tablet. The terminology used for the species descriptions follows Baba et al. (2009) and Baba et al. (2011). Size is indicated by the carapace length excluding rostrum or postorbital carapace length (PCL). Rostrum length is measured as the straight-line distance from the base (frontal margin) to the distal tip of the rostrum; rostrum width is taken as the straight-line distance at the rostrum’s base width (between frontal margin). Measurements of appendages are taken on dorsal (pereopod 1), lateral (pereopods 2–4 and maxilliped 3) midlines. Ranges of morphological and meristic variations are included in the descriptions. Abbreviations used in the descriptions are indicated as follows: **Mxp** = maxilliped; **P1** = pereopod 1 (cheliped); **P2–4** = pereopods 2–4 (walking legs 1–3); **M** = male; **F** = female; **ov.** = ovigerous. The examined specimens are deposited in the Smithsonian National Museum of Natural History (**USNM**), Washington DC and Invertebrate Zoology collection in the Museum of Comparative Zoology (**MCZ:IZ**), Harvard, University, Cambridge, MA. Some specimens previously deposited at the Florida International Crustacean Collection (**FICC**), Florida International University, Miami, FL (**FIU-HBG**); and University of Louisiana at Lafayette’s Zoological Collection (**ULLZ**), were recently donated to the USNM and MCZ:IZ collections.

### ﻿Micro-computed tomography

The specimens selected for 3D imaging were mounted in plastic vials filled with ethanol and secured using synthetic cotton and/or parafilm to keep them stationary during the scanning process. The vials were sealed using parafilm to avoid evaporation.

The micro-computed tomography (micro-CT) scan was performed using a SkyScan 1273 scanner (Bruker MicroCT, Kontich, Belgium) located at Digital Imaging Facilities (DIF), MCZ. The scanner was equipped with a Hamamatsu 130/300 tungsten x-ray source, 40–130 kV, and a flat-panel x-ray detector with 6-megapixel (3072 × 1944). We chose the following scanning parameters: source current = 100 µA, source voltage = 75 kV, exposure time = 1,000 ms, frames averaged = 3–4, rotation step = 0.2, frames acquired over 180° = 960, filter = no, binning = no, flat field correction = activated. The scanning time ranged between 50 and 120 min. The reconstruction of the scans was processed using the software NRecon 1.6.6.0 (Bruker MicroCT, Kontich, Belgium). To enhance image contrast and compensate for the ring and streak artifacts, the following reconstruction parameters were set: smoothing = no, ring artifact correction = 5–11, and beam hardening correction = activated. 3D renderings of micro-computed tomography x-ray images were performed using Amira software (Bruker MicroCT, Kontich, Belgium).

### ﻿Molecular analyses

The workflow for DNA extraction, amplification of the cytochrome *c* oxidase subunit (*COI*) followed Rodríguez-Flores et al. (2024). DNA was extracted with the DNeasy Blood and Tissue kit (Qiagen), following the manufacturer’s protocol after overnight digestion. For the amplification by PCR, the primers LCOI 1490 and HCO2198 were used (Machordom et al. 2003). Thermal conditions followed Rodríguez-Flores et al. (2023), with an annealing temperature ranging from 45 °C to 50 °C. DNA extraction, amplification and Sanger sequencing were done in the Laboratories of Analytical Biology (LAB) located at the Smithsonian NMNH. Genetic distances were calculated with the software Genious Prime 2024 Build 2024-04-12 19:00 (https://www.geneious.com/), including closely related species with genetic data available (Rodríguez-Flores et al. 2023).

## ﻿Results

### ﻿Taxonomy

**Infraorder Anomura**

**Superfamily Galatheoidea**

**Family Munidopsidae**

**Genus *Munidopsis* Whiteaves, 1874**

#### ﻿ Munidopsis messingi

Taxon classificationAnimaliaDecapodaMunidopsidae

sp. nov.

27F7770D-F67B-500B-A2FA-4F5C4D8F546B

https://zoobank.org/36384BE1-9FC9-4807-9B42-C09955195B04

**Figure 2.**
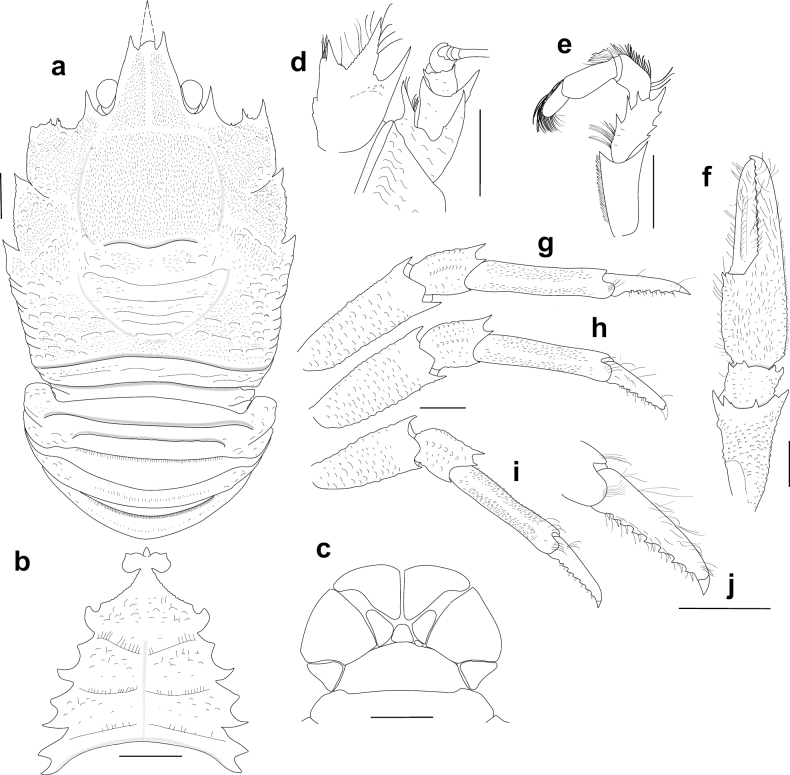
*Munidopsismessingi* sp. nov., M 11.5 mm PCL, holotype (USNM 1424972, CURI 16056), Curaçao. A. Carapace and abdomen, dorsal view; B. Sternal plastron; C. Telson; D. Cephalic region, showing antennular and antennal peduncles, ventral view; E. Left Mxp3, lateral view; F. Right P1, dorsal view; G. Right P2, lateral view; H. Right P3, lateral view; I. Right P4, lateral view; J. Right P4 dactyli, lateral view. Scale bars: 2 mm.

**Figure 3.**
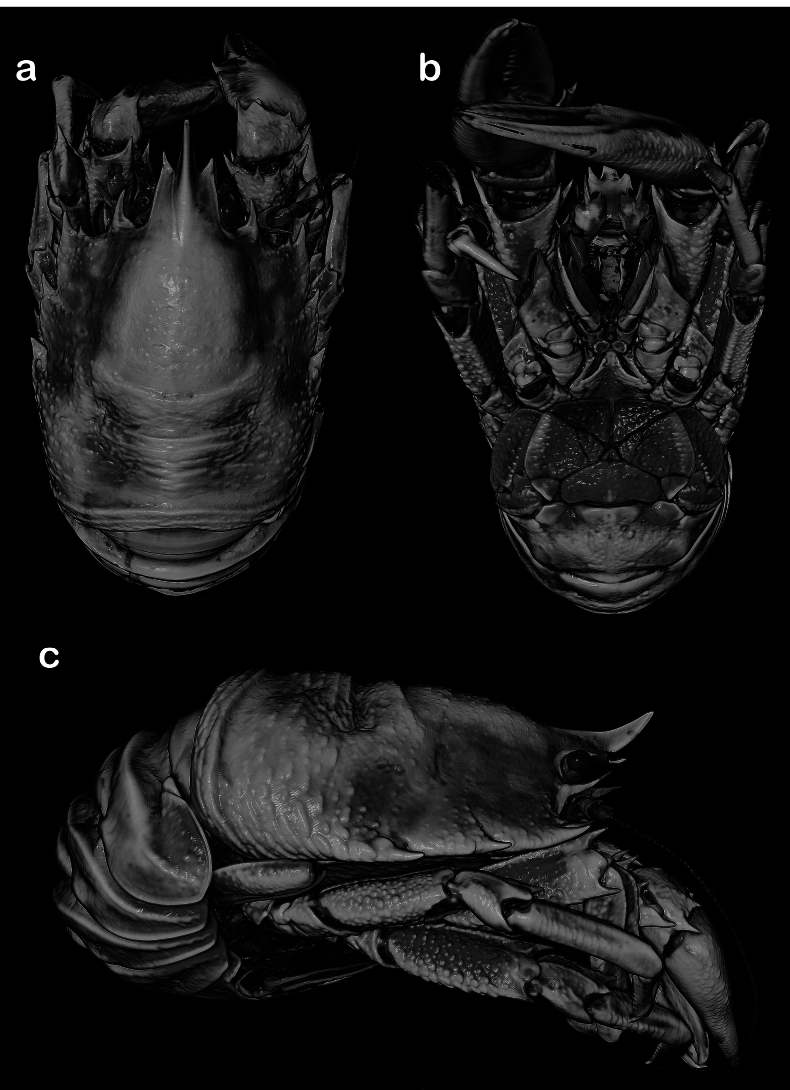
*Munidopsismessingi* sp. nov., 3D rendering of micro-CT images, 23.5 mm PCL, paratype (: IZ 172998, HBG 9802) east coast of Florida: A. dorsal B. ventral; C. lateral views.

**Figure 4.**
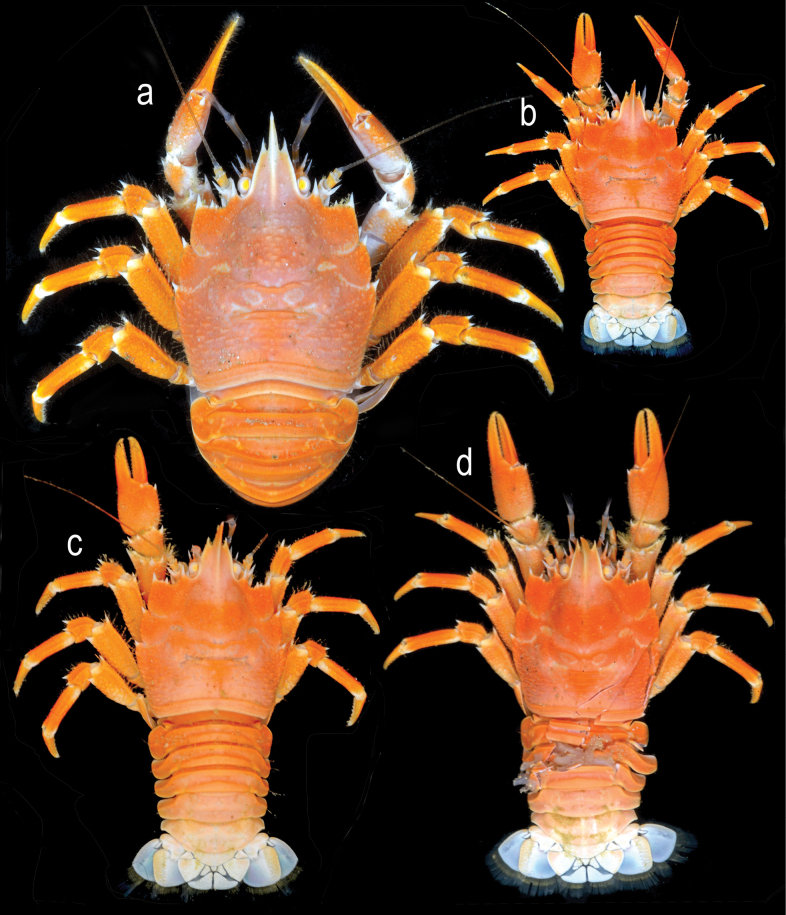
*Munidopsismessingi* sp. nov., color images of fresh specimens, 1 M 10.9 mm PCL, paratype (USNM 1554267 [CURI 17020]), Curaçao: A, B. M 13.0 mm PCL, paratype (USNM1406053 [ULLZ 15927; CURI 14047]), Curaçao, C. M 9.2 mm PCL paratype (USNM 1406056 [ULLZ 15933; CURI 14055]), Curaçao, D. A. Dorsal habitus, abdomen flexed; B. Dorsal habitus, abdomen extended; C. Dorsal habitus, damaged abdomen extended; D. Dorsal habitus, abdomen extended. Photographs by DLF.

Figs 1 , 2A–J , 3A–C , 4A–D

##### Material examined.

***Holotype*.** • North Atlantic Ocean, Curaçao, DROP, 2016, CURI 16056, Curaçao Sea Aquarium, east of downline, Bapor Kibra, 12.082254 -68.897365, 253–264 m, 14 October 2016: M 11.5 mm (USNM 1424972, GenBank Accession No. PV297963). ***Paratypes*.** • North Atlantic Ocean, Atlantic coast of Florida, 27.07707, -79.319214, no depth or date data: 1 ov. F 23.5 mm (MCZ:IZ 172998, ex-HBG 9802), GenBank Accession No. PV297964). • Curaçao, DROP, 2012, CURI 12032, off of Substation Curaçao downline, 12.040327, -68.781043, no depth data, 29 May 2012: 1 F 19 mm (USNM 1198913). • CURI 11529, Headed East out of gate/substation, no locality data, 173–259 m, 27 May 2011: 1 F 18.5 mm (USNM 1160002). • CURI 14047, East at Substation Curaçao downline, 215–309 m, 12.083197, -68.899058, 23 September 2014: 1 M 13.0 mm (ULLZ 15927, USNM 1406053). • CURI 14055, off of Substation Curaçao downline, 12.083197, -68.899031, 142–280 m, 24 September 2014: 1 M 9.2 mm (ULLZ 15933, USNM 1406056). • CURI 17020, Curaçao Sea Aquarium east of downline, Bapor Kibra, 271 m, 12.0823, -68.8973, 2 November 2017: 1 M 10.9 mm (USNM 1554267, GenBank Accession No. PV810825).

##### Etymology.

Named after Charles “Chuck” Messing, recently deceased, in honor of his dedication to and passion for ocean exploration, and his “larger than life” entertaining and kind personality. This is in recognition of his significant contributions to the biology, natural history and taxonomy of echinoderms, crustaceans and other invertebrates.

##### Diagnosis.

Carapace dorsally covered with short setae, pilose, gastric, hepatic and anterior branchial areas smooth, posterior branchial area with short scales or scattered granules (Fig. 3A–C). Rostrum moderately broad, dorsally elevated and with median carina, distally trifid. Frontal margin slightly concave behind ocular peduncle. Orbit excavated, orbital angle above antennal peduncle produced into sharp spine. 1–3 small spines close to anterolateral angle. Anterolateral spine strong. Branchial margin armed with 2 strong spines. Abdominal tergites unarmed. Telson divided into 10 plates. Sternite 3 anterolaterally rounded, anterior margin with median lobe flanked by 2 lobes, sternite 4 broadly subtriangular. Eyes unarmed, movable, epistomial spine present, cornea globular, elongated. Article 1 of antennule with dorsolateral and distolateral spines. Article 1 of antenna with strong distomesial spine and distolateral spines. Mxp3 merus with 3 strong spines on flexor margin, small distal spine, extensor margin with 4 spines. P1 stout, length less than twice PCL; meri and carpi armed with distal spines; palm and fingers unarmed; fixed finger without denticulate carina on distolateral margin; dactylus dorsally carinate. P2–4 stout; meri carinate on dorsal margin; dactyli slender, distally curving, flexor margin with 6–8 small teeth decreasing proximally along entire length. Epipods present on P1 and 2.

##### Description.

***Carapace***: Slightly broader than long, widest at midlength; moderately convex from side to side. Dorsal surface pilose; gastric, hepatic and anterior branchial areas smooth, densely covered with short setae; posterior branchial area with short scales or scattered granules (Figs 2A, 3A, C); cardiac and intestinal regions covered with larger scales, each scale with few short setae. Regions well delineated by deep furrows including distinct anterior and posterior cervical grooves. Gastric region slightly elevated. Cardiac region divided by a transverse furrow in anterior and posterior cardiac regions. Posterior margin unarmed, preceded by elevated ridge. Rostrum moderately broad, dorsally elevated, width 0.2–0.3 × anterior width of carapace, directed strongly upwards, dorsally carinate, distally trifid, with strong median spine twice as long as lateral spines (Fig. 2A), 0.4 × PCL, 1.3–1.8 × as long as broad. Frontal margin concave behind ocular peduncle, orbital angle above antennal peduncle produced into sharp spine, reaching or surpassing distal margin of cornea; spine below antennal proximal mesial angle, ventral to frontal margin, close to epistomial region; 1–3 small spines close to anterolateral spine. Lateral margins slightly convex, converging posteriorly; anterolateral spine sharp; anterior branchial margin with 1 strong spine; 1 strong branchial spine just behind posterior branch of cervical groove. Pterygostomian flap surface covered with granules and scales, anteriorly narrowly rounded.

***Sternum***: 0.9 × as long as broad, maximum width at sternite 7. Sternite 3 broad, 3.0 × wider than long, anterolaterally rounded, anterior margin with median lobe flanked by 2 subacute lobes. Sternite 4 narrowly elongated anteriorly; surface depressed in midline, with scattered short scales; greatest width 2.3 × that of sternite 3, 1.7 × wider than long.

***Pleon***: Unarmed; tergite 2 with 2 elevated transverse ridges, lateral parts of dorsal surfaces covered with granules and scales; tergites 3–6 with only anterior; tergite 6 with weakly produced posterolateral lobes and nearly transverse posteromedian margin. Telson composed of 10 plates, 1.5 × as wide as long.

***Eye***: Eyestalk movable, partially concealed by rostrum; peduncle with few granules, shorter than cornea length; cornea, elongated, longer than peduncle, ovoid; lateral surface contiguous to epistomial spine, epistomial spine ventral to frontal margin.

***Antennule***: Article 1 of peduncle with granules on anterolateral ventral surface, armed with subequal dorsolateral and distolateral spines; distomesial margin with strong spine.

***Antenna***: Peduncle slightly exceeding eye. Article 1 with strong distomesial and distolateral spines, each surpassing distal margin of article 2. Article 2 with distomesial and distolateral spines, distolateral spine much stronger. Article 3 armed with small distomesial and distolateral spines. Article 4 unarmed.

***Mxp3***: Lateral surface with few granules. Ischium as long as merus measured on extensor margin merus with 3 strong spines subequal in size and 1 smaller distal spine on flexor margin; extensor margin with 4 spines, distal spine strongest.

***P1***: Stout, with numerous minute granules and short scales, each scale marginally with few short setae, 1.3 × longer than PCL. Merus 2.2 × carpus length, distally with stout spines. Carpus 0.9 × longer than broad, with some short distal spines, few acute granules on dorsal surface. Palm unarmed, stout, slightly longer than carpus, 1.2 × longer than broad. Fingers unarmed, 1.2 × longer than palm, opposing margins nearly straight, not gaping, spooned; fixed finger without denticulate carina on distolateral margin, mobile finger with dorsal carina.

***P2–4***: Stout, pilose, with short scales and granules, cylindrical in cross-section, slightly decreasing in size posteriorly. P2 merus stout, overreaching tip of P1, 0.6 × carapace length, 2.7 × longer than high, equal to 1.1 × length of P2 propodus. P2–4 meri decreasing in length posteriorly (P3 merus 0.9 × length of P2 merus, P4 merus 0.85 × length of P3 merus); extensor margin carinate, with small granules along entire length, distal part flattish ending in thick spine; flexor margin granulate; P2–4 carpi each with 1 thick distal spine on extensor margin, lateral surface with granulated carina; P2–4 propodi 4.0–5.0 × as long as high, triangular in cross-section, unarmed. P2–4 dactyli 0.5 × length of propodi; distal claw short, moderately curved; flexor margin distally curved, with 6–8 dactylar teeth on entire length, each tooth with slender corneous spine, ultimate tooth closer to penultimate tooth than to dactylar angle.

***Epipods***: Present on P1 and 2.

***Coloration***: Carapace, P1–4 and chelipeds entirely light to reddish orange. Distal parts of rostrum, articles and spines whitish (Fig. 4A–D).

##### Ecology.

Unknown.

##### Distribution.

Curaçao and East coast of Florida; 173–264 m.

##### Remarks.

This species belongs to a group of species having a trifid and elevated rostrum, an unarmed carapace and abdomen, 8–10 telson plates, a stout P1 that is less than twice the PCL, stout P2–4, and epipods on P1 and 2. In addition to *M.messingi* sp. nov., this species group includes *M.expansa* Benedict, 1902 from the western Atlantic and *M.testuda* Rodríguez-Flores, Seid, Rouse & Giribet, 2023, known only from the Galapagos Islands in the eastern Pacific. The new species can be distinguished from the aforementioned two species by the following morphological characters:

1. The carapace gastric, hepatic and branchial regions are covered with granules and scales in *M.expansa* and *M.testuda*, but these areas are covered with dense pilosity in the new species.
2. The carapace anterolateral angle is produced as a broad spine in *M.testuda* and *M.expansa*, whereas the spine is sharp, much longer and preceded by a cluster of 1–3 spines in the new species.
3. The carapace excavate orbit in the new species terminates laterally in a sharp, long spine, sometimes surpassing the distal margin of the cornea, but this spine is absent in *M.expansa* and *M.testuda.*
4. The lateral margin of the rostrum is concave in *M.testuda*, slightly convex in the new species and divergent in *M.expansa*.
5. The distomesial angle of the antennular peduncle article 1 is armed with a distinct spine in the new species, whereas it is unarmed in *M.expansa* and *M.testuda*.
6. The new species differs from *M.expansa* and *M.testuda* in having a much longer distolateral spine of the antennal peduncle article 1.
7. The flexor margin of Mxp3 merus is unarmed in *M.expansa*, whereas it is armed with 3 distinct spines in the new species.
8. The flexor margin of P2–4 dactyl is armed with 6–8 teeth in the new species but has 11–12 teeth *M.expansa* and *M.testuda*.

Genetic divergence for the *COI* marker ranged from 14.0 to 15.6% between the new species and its closely related congeners (see Table 1 for genetic distances).

**Table 1.** Average genetic distances (p-distances) for the *COI* marker calculated for each species, including *Munidopsistestuda* Rodríguez-Flores, Seid, Rouse & Giribet, 2023.

	Munidopsisexpansa	Munidopsistestuda
*Munidopsisexpansa*		
*Munidopsistestuda*	0.107	
*Munidopsismessingi* sp. nov.	0.156	0.140

#### ﻿ Munidopsis expansa

Taxon classificationAnimaliaDecapodaMunidopsidae

Benedict, 1902

50C8FCA4-43B5-5458-8D43-783FC14EF903

**Figure 5.**
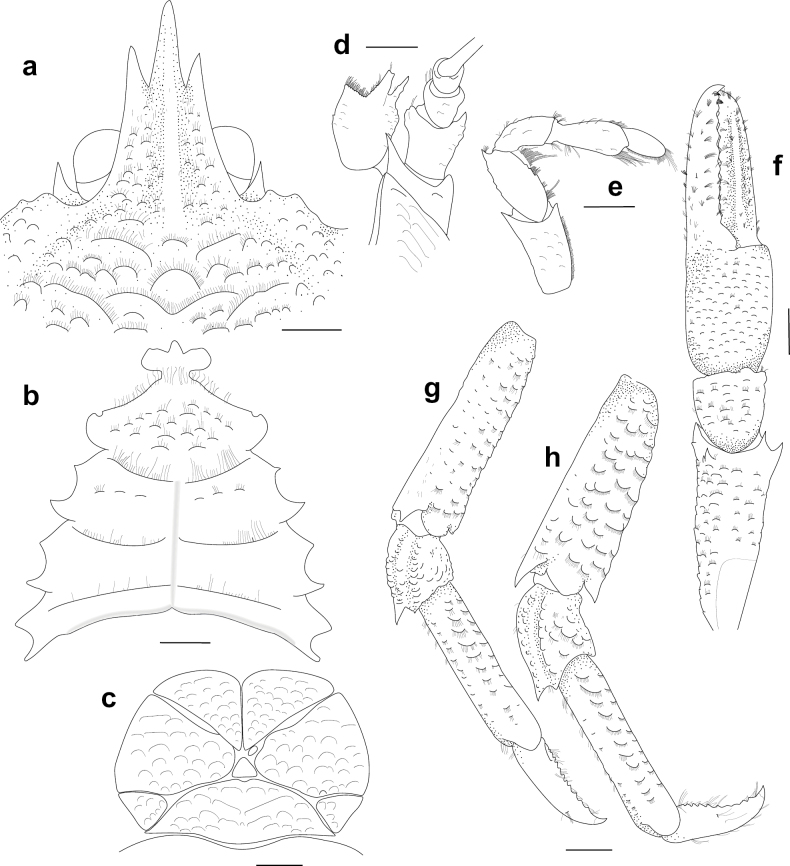
*Munidopsisexpansa* Benedict, 1902, ov. F 21 mm PCL, holotype (USNM 20561), Florida. A. Anterior part of carapace, dorsal view; B. Sternal plastron; C. Telson; D. Cephalic region, showing antennular and antennal peduncles, ventral view; E. Right Mxp3, lateral view; F. Left P1, dorsal view; G. Left P2, lateral view; H. Left P3, lateral view. Scale bars: 2 mm.

**Figure 6.**
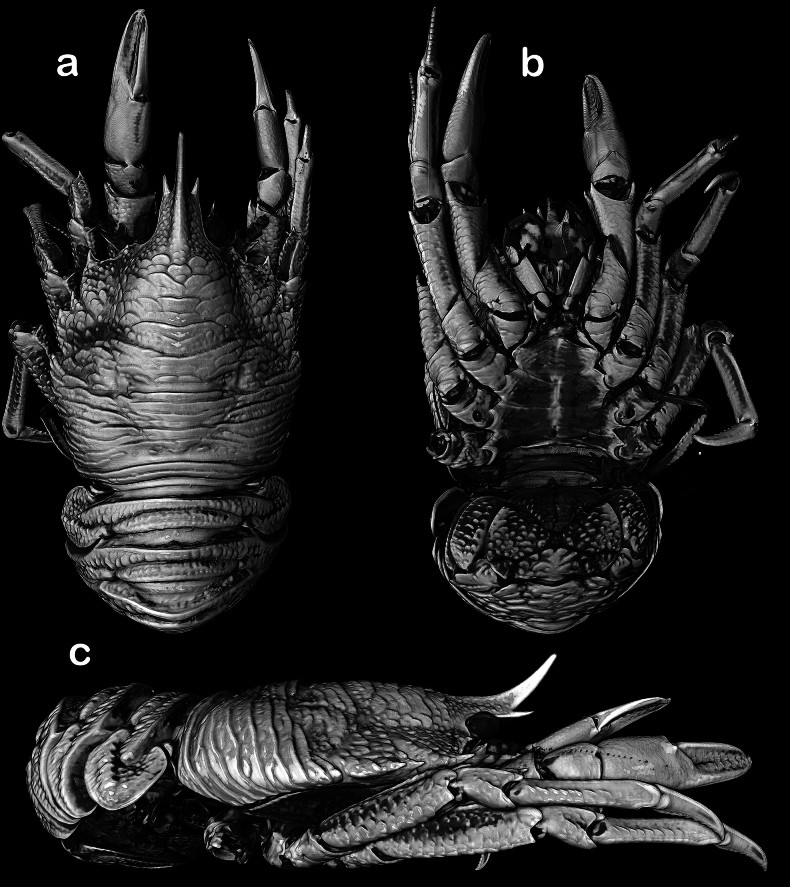
*Munidopsisexpansa* Benedict, 1902, 3D rendering of micro-computed tomography x-ray images, F 17.7 mm PCL (MCZ CRU 11732), Cuba: A. Dorsal; B. Ventral; C. Lateral views.

**Figure 7.**
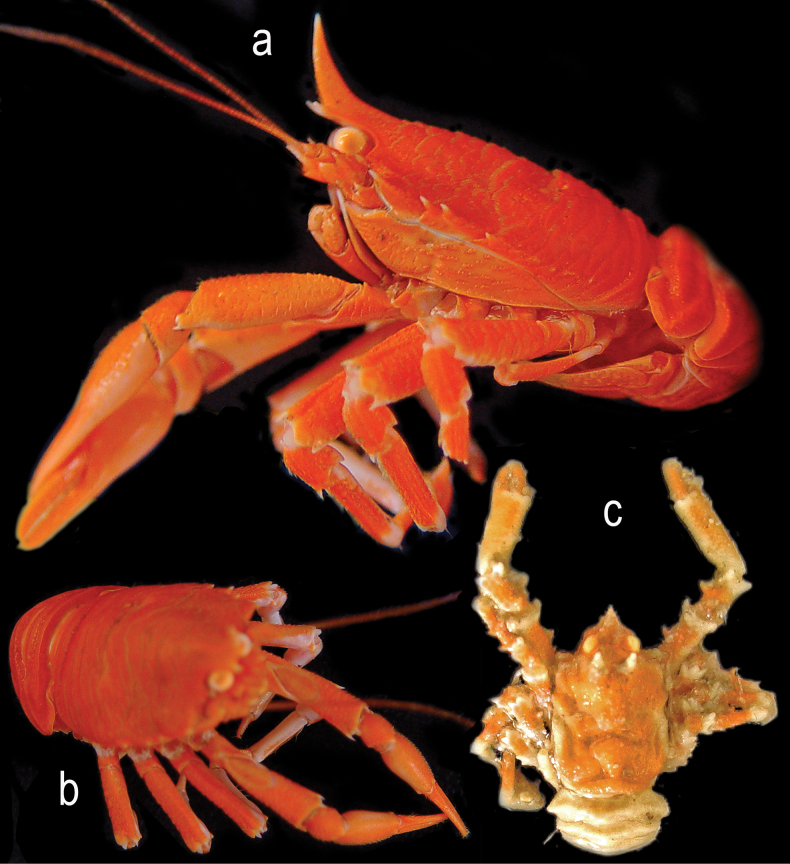
Color images of fresh specimens: *Munidopsisexpansa* Benedict, 1902, (USNM 1194614) West Florida slope: A. Lateral habitus; B. dorsal habitus, abdomen flexed; C. *Munidopsisturgida* Rodríguez-Flores, Macpherson & Machordom, 2018, (USNM 1666812) dorsal habitus, abdomen flexed. Photographs by MN (C) and C. Morrison (A, B).

Figs 1 , 5A–H , 6A–C , 7A, B

Munidopsis expansa Benedict, 1902: 282, fig. 26 (type locality: off Florida, 770 m). — Chace 1942: 81. — Pequegnat and Pequegnat 1970: 147 (no record). — Pequegnat and Pequegnat 1971: 19. — Baba et al. 2008: 141 (compilation). — Felder et al. 2009: 1066 (compilation).

##### Material examined.

***Holotype*.** • North Atlantic Ocean, United States, off Florida, Daytona Beach, 29.65, 79.8167, 770 m, 4 May 1886: ov. F 21.0 mm (USNM 20561).

##### Other material.

• West Florida Slope, USGS-LOPH II-ROV-2010-CH-008, 26.207882, -84.725818, 508–492 m, 29 September 2010: 1 M 16.0 mm (USNM 1194614, GenBank Accession No. PV297962), USGS-LOPH II-CH-2010-081, 26.213611, -84.725486, 525 m, 30 September 2010: 1 F 17.1 mm (USNM 1194615, GenBank Accession No. PV297961). • North Atlantic Ocean, Gulf of Mexico, United States, R/V OREGON II, Station 11140, 24.25, -87.73, 503 m, 10 August 1970: not measured (USNM 1662589). • North Atlantic Ocean, Florida Straits/Gulf of Mexico, Cuba, off Havana, RV Atlantis, Harvard-Havana Expedition, 23.0750000, 82.616666, 603.5 m, 23 March 1939: 1 F 17.7 mm (MCZ CRU 11732). • Belize, East of Stann Creek, 16.97, -87.88, 457–732 m, 10 June 1962: 1 F 19.0 mm (USNM 268733).

##### Diagnosis.

Carapace dorsally covered with scales, shorter and more abundant on gastric region, wider scales posteriorly; cervical grooves distinct (Fig. 6A–C). Rostrum moderately broad, dorsally elevated and with median carina, distally trifid. Frontal margin slightly concave behind ocular peduncle. Orbit slightly excavated, outer orbital angle with small blunt lobe. Anterolateral angle armed with broad spine. Branchial margin armed with 3 broad spines. Abdominal tergites unarmed. Telson divided into 8 plates. Sternite 3 anterolaterally rounded, anterior margin with median lobe flanked by 2 lobes, sternite 4 broadly subtriangular. Eyes unarmed, movable, epistomial spine present, cornea globular, elongated. Article 1 of antennule with dorsolateral and distolateral spines. Article 1 of antenna with strong distomesial spine and distolateral spines. Mxp3 merus serrated but without distinct spines on flexor margin, extensor margin with short spine. P1 stout, length less than twice PCL; meri armed with distal spines; carpi, palm and fingers unarmed; fixed finger without denticulate carina on distolateral margin; dactylus dorsally carinate. P2–4 stout; meri carinate on dorsal margin; dactyli slender, distally curving, flexor margin with 11–12 teeth decreasing proximally along entire length. Epipods present on P1 and 2.

##### Redescription.

***Carapace***: Slightly broader than long, widest at midlength; moderately convex from side to side. Dorsal surface densely covered with scales, most abundant on gastric region, each scale marginally with short setae; hepatic and anterior branchial regions with scales and some granules; posterior cardiac and intestinal region covered with larger scales. Regions well delineated by deep furrows including distinct anterior and posterior cervical grooves. Gastric region slightly elevated. Cardiac region divided by a transverse furrow in anterior and posterior cardiac regions. Posterior margin unarmed, preceded by elevated ridge. Rostrum moderately broad, dorsally elevated, with median carina, width 0.2–0.3 × anterior width of carapace, directed strongly upwards, distally trifid, with strong median spine twice as long as broad lateral spines (Fig. 5A), 0.4 × PCL, 1.3–1.8 × as long as broad. Frontal margin slightly concave behind ocular peduncle; blunt outer orbital angle above antennal peduncle, sometimes armed with 1 or 2 minute spines (absent in the holotype); spine below antennal proximal mesial angle, ventral to frontal margin, close to epistomial region. Lateral margins slightly convex, converging posteriorly; anterolateral spine broad; anterior branchial margin with 2 broad spines; 1 broad branchial spine just behind posterior branch of cervical groove. Pterygostomian flap surface covered with granules and scales, anteriorly rounded.

***Sternum***: 0.9 × as long as broad, maximum width at sternite 7. Sternite 3 broad, 1.5 × wider than long, anterolaterally rounded, anterior margin with median lobe flanked by 2 rounded lobes. Sternite 4 narrowly elongated anteriorly; surface depressed in midline, with scattered scales; greatest width 2.3 × that of sternite 3; 2 × wider than long.

***Pleon***: Unarmed; tergites 2–4 each with 2 elevated transverse ridges, lateral part of dorsal surface covered with granules and scales; tergites 5 and 6 with anterior ridge only; tergite 6 with weakly produced posterolateral lobes and nearly transverse posteromedian margin. Telson composed of 8 plates; 1.4–1.5 × as wide as long.

***Eye***: Eyestalk movable, partially concealed by rostrum; peduncle covered with few granules; cornea elongated, longer than peduncle, ovoid; lateral surface contiguous to epistomial spine, epistomial spine ventral to frontal margin.

***Antennule***: Article 1 of peduncle with subequal dorsolateral and distolateral spines, dorsolateral spine slenderer; distomesial margin slightly produced, granulated.

***Antenna***: Peduncle slightly exceeding eye. Article 1 with strong distomesial and distolateral spines, not reaching distal margin of article 2, distolateral spines shorter. Article 2 with well-developed distolateral spine, distomesial margin produced, granulated. Articles 3 and 4 unarmed.

***Mxp3***: Lateral surface with granules. Ischium as long as merus measured on extensor margin. Merus serrated, but without distinct spines on flexor margin; extensor margin weakly serrated, with small distal spine.

***P1***: Stout, with numerous minute granules and scales, each scale marginally with few short setae; 1.2–1.6 × PCL. Merus 2.6 × carpus length, with 4 distal spines. Carpus 0.7–0.9 × longer than broad, unarmed, with few granules on dorsal and lateral surfaces. Palm unarmed, stout, 2 × carpus length, 1.2 × longer than broad. Fingers unarmed, 1.2 × longer than palm, opposing margins nearly straight, not gaping, spooned; fixed finger without denticulate carina on distolateral margin, mobile finger with dorsal carina.

***P2–4***: Stout, coarsely granulated, devoid of setae, cylindrical in cross-section, slightly decreasing in size posteriorly. P2 merus stout, not surpassing P1 length, 0.6 × PCL, 3.5 × longer than high, 1.1 × length of P2 propodus. P2–4 meri decreasing in length posteriorly (P3 merus 0.9 × length of P2 merus, P4 merus 0.85 × length of P3 merus); extensor margin carinate, with small granules along entire length, distally flattish, ending in thick spine; flexor margin granulated. P2–4 carpi with thick distal spine on extensor margin, lateral surface with granulated carina. P2–4 propodi 3.5–5 × as long as high, triangular in cross-section, unarmed. P2–4 dactyli 0.5–0.6 × length of propodi; distal claw short, flexor margin nearly straight, with 11 or 12 dactylar teeth on entire length, each tooth with slender corneous spine, ultimate tooth closer to penultimate tooth than to dactylar angle.

***Epipods***: Present on P1 and 2.

***Coloration***: Bright orange. Distal parts of articles, rostrum and tips of spines whitish (Fig. 7A, B).

##### Ecology.

Specimens collected on the West Florida Slope, at station USGS-LOPH II-ROV-2010-CH-008 were observed walking on dead coral rubble of *Desmophyllumpertusum*. In general, the habitat was characterized by large, mostly dead colonies of *D.pertusum* usually found on the edge of outcrops. Smaller live colonies of *D.pertusum*, *Plummerella* sp., as well as other octocoral species and hexactinellid sponges, were found scattered over dead, consolidated coral rubble.

##### Remarks.

*Munidopsisexpansa* was initially described by Benedict (1902) from material collected off Florida. This species belongs to a group of species characterized by a trifid and elevated rostrum, unarmed carapace and abdomen, 8–10 telson plates, stout P1 (less than twice PCL), stout P2–4 and epipods on P1 and 2. In addition to *M.expansa* from the western Atlantic, this group of species includes *M.testuda* Rodríguez-Flores, Seid, Rouse & Giribet, 2023, known only from the Galapagos Islands of the eastern Pacific, and the new species described herein. Differences among these species are discussed under the Remarks of the new species.

##### Distribution.

North coast of Cuba, Gulf of Mexico, Atlantic coast of Florida; 457–1107 m.

#### ﻿ Munidopsis turgida

Taxon classificationAnimaliaDecapodaMunidopsidae

Rodríguez-Flores, Macpherson & Machordom, 2018

C1C55D32-24D7-5CD8-84F1-ECA25BFDC969

Figs 1 , 7C , 8A, B

Munidopsis turgida Rodríguez-Flores et al., 2018: 576, fig. 4 (type locality: Guadeloupe 457–484 m).— Rodríguez-Flores et al. 2023: figs 1, 2 (molecular data).

##### Material examined.

• North Atlantic Ocean, West Florida Slope, USGS-LOPH II-JSLII-09-GOM-3722, 26.2041431, 84.7271528, 536–500 m, 6 September 2009: 1 M 5.1 mm (USNM 1666812). • USGS-LOPH II-CH-2010-081, 26.213611, 84.725486, 525 m, 30 September 2010: 1 F not measured (USNM 1704796, GenBank Accession No. PV297965).

##### Diagnosis.

**Figure 8.**
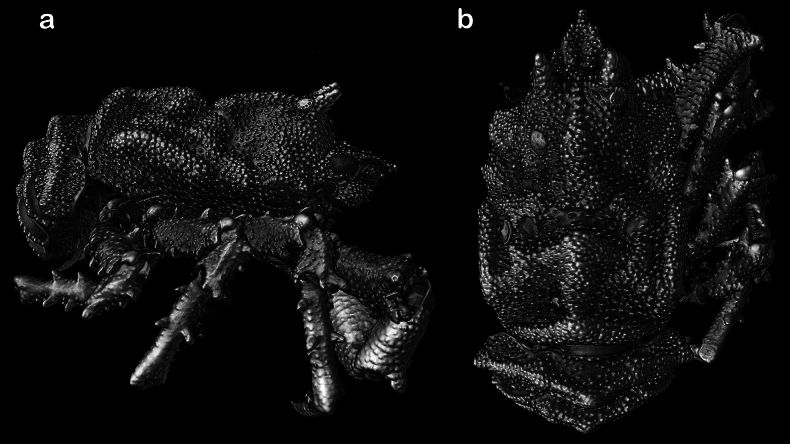
*Munidopsisturgida* Rodríguez-Flores, Macpherson & Machordom, 2018, 3D rendering of micro-CT images, M 5.1 mm (USNM 1666812), West Florida slope: A. Dorsal B. Ventral lateral views.

Carapace and abdomen densely covered with rounded granules (Fig. 8A, B), slightly longer than broad; with 2 epigastric protuberances; dorsal regions well delimited by furrows. Rostrum spade-shaped in dorsal view, constricted between eyes. Frontal margin slightly concave behind ocular peduncle, transverse between antenna and anterolateral angle of carapace. Lateral margins unarmed; subparallel, anterolateral corner rounded. Sternum longer than wide, maximum width at sternite 6 or 7; sternite 3 moderately broad, twice wider than long, width about half that of sternite 4; anterior margin of sternite 4 widely triangular. Abdomen unarmed; telson composed of 8 plates. Eyes small, fused to rostrum, without eye-spines; granulate overgrowths covering proximo-mesial part of cornea dorsally and ventrally; cornea globular. Mxp3 merus with granules on flexor margin. P1 more than twice PCL. P2–4 stout, with protuberances; extensor margins of articles not cristate; propodi not expanded distally; dactyli moderately curved distally, flexor margin with teeth decreasing in size proximally, each tooth with slender corneous spine. P2 not reaching end of P1. Epipods on P1.

***Coloration***: Carapace and chelae light orange. Abdomen white. P1–4 orange with white bands. Spines on P1–4 and carapace epigastric protuberances white (Fig. 7C).

##### Ecology.

The specimen collected on the West Florida Slope at station USGS-LOPH II-JSLII-09-GOM-3722 was observed on live *Desmophyllumpertusum*. In general, the habitat was characterized by discrete, relatively large colonies of both live and dead *D.pertusum*, interspersed with areas of coral rubble and soft sediments.

##### Remarks.

The specimen sequenced from the Gulf of Mexico is genetically identical to the Caribbean holotype (0% of molecular divergence) for the *COI* fragment obtained (Table 1).

##### Distribution.

Known only from the type locality in Guadeloupe and the present new record from the Gulf of Mexico, West Florida Slope; 457 to 536 m depth.

## ﻿Discussion

Using an integrative approach, including morphological data, micro-CT scanning evidence, and molecular analyses, we have increased our knowledge of squat lobster biodiversity in the Gulf of Mexico and the Caribbean Sea.

Integrative taxonomy, defined as the integration of independent lines of evidence in taxonomy (Pante et al. 2015), has become the standard approach for describing new species of all taxa, usually by combining molecular data with an exhaustive morphological examination (e.g., Cobo et al. 2024; Corbari et al. 2024). Many recent studies on squat lobster taxonomy incorporate molecular data into a comprehensive workflow. Taxonomic revisions and redescriptions of previously described species, many of which lack modern illustrations or diagnoses that meet current taxonomic standards, are improved significantly using an integrated methodology (Dong et al. 2021; Rodríguez-Flores et al. 2023; 2024). The use of novel techniques such as 3D micro-CT morphological scans has proven invaluable for detecting micro-ornamentation patterns that might otherwise go unnoticed with traditional methods, providing new morphological characters for both known and newly described species. Furthermore, such imaging reduces the need for excessive handling and shipping of type specimens, minimizing the risk of damage to these often fragile, older specimens (e.g., Ng et al. 2021; Chen and Chan 2023; Rodríguez-Flores and Schnabel 2023).

Despite having been the focus for many previous surveys of crustacean diversity, the Gulf of Mexico and Caribbean still harbor undiscovered species. The species analyzed in this study are endemic to the western Atlantic and include rare taxa known only from the holotype or a few additional records (Baba et al. 2008; Rodríguez-Flores et al. 2018). The disjunct distributions observed for *Munidopsismessingi* sp. nov. and *M.turgida*, suggest that the Gulf of Mexico and the Caribbean Sea remain undersampled. Given that the number of undiscovered species may be higher than previously estimated (De Grave et al. 2023), continued collecting efforts and comprehensive taxonomic studies are essential to fully assess the biodiversity of the deep sea in this region.

## ﻿Conclusion

The present study reports key taxonomic findings from recently collected squat lobsters in the western Atlantic. A new species of *Munidopsis* was identified—from Curaçao and off eastern Florida—closely related to *M.expansa*, which was also re-described with new morphological data. Additionally, a specimen of *M.turgida*, previously known only from Guadeloupe, was found in the Gulf of Mexico, extending its known range. These findings are supported by morphological features (including micro-CT imagery) and *COI* barcode sequences, contributing to a better understanding of squat lobster diversity in the region and facilitating species identification through eDNA.

## Supplementary Material

XML Treatment for Munidopsis messingi

XML Treatment for Munidopsis expansa

XML Treatment for Munidopsis turgida

## ﻿Additional information

### Use of AI

No use of AI was reported.

### Author ORCIDs

Paula C. Rodríguez-Flores https://orcid.org/0000-0003-1555-9598

Heather D. Bracken-Grissom https://orcid.org/0000-0002-4919-6679

Rafael Lemaitre https://orcid.org/0000-0003-2828-612X

### Data availability

All of the data that support the findings of this study are available in the main text or Supplementary Information.
